# Three-Dimensional Hierarchical Porous Structure of PPy/Porous-Graphene to Encapsulate Polysulfides for Lithium/Sulfur Batteries

**DOI:** 10.3390/nano8080606

**Published:** 2018-08-09

**Authors:** Yongguang Zhang, Zhumabay Bakenov, Taizhe Tan, Jin Huang

**Affiliations:** 1 School of Materials and Energy, Synergy Innovation Institute of GDUT (Heyuan), Guangdong University of Technology, Guangzhou 510006, China; ygzhang126@126.com; 2 Institute of Batteries LLC, National Laboratory Astana, School of Engineering, Nazarbayev University, 53 Kabanbay Batyr Avenue, Astana 010000, Kazakhstan; zbakenov@nu.edu.kz

**Keywords:** lithium/sulfur battery, hierarchical 3D porous structure, sulfur/polypyrrole/porous graphene composite

## Abstract

Herein, we demonstrate the fabrication of a three-dimensional (3D) polypyrrole-coated-porous graphene (PPy/PG) composite through in-situ polymerization of pyrrole monomer on PG surface. The PPy/PG displays a 3D hierarchical porous structure and the resulting PPy/PG hybrid serves as a conductive trap to lithium polysulfides enhancing the electrochemical performances. Owing to the superior conductivity and peculiar structure, a high initial discharge capacity of 1020 mAh g^−1^ and the reversible capacity of 802 mAh g^−1^ over 200 cycles are obtained for the S/PPy/PG cathode at 0.1 C, remaining the remarkable cyclic stability. In addition, the S/PPy/PG cathodes demonstrate an excellent rate performance exhibiting 477 mAh g^−1^ at 2 C.

## 1. Introduction

Recently, lithium-ion batteries (LIBs) are being widely used in portable electronic devices and considered for future hybrid electric vehicles due to their high energy density and excellent cyclic performance. However, traditional LIBs with transition metal oxides cathodes and graphite-based anodes cannot meet the demand of the market due to the limited capacity of electrode materials for LIBs. Therefore, a considerable research effort has been focused on developing novel electrode materials with high theoretical specific capacity that can maximize the energy density of LIBs [1,2]. Furthermore, lithium/sulfur (Li/S) batteries exhibit significant advantages, such as the natural abundance and eco-friendly nature of sulfur, and high theoretical specific capacity of 1672 mAh g^−1^. The energy density of 2600 Wh kg^−1^ for Li/S batteries is five times larger than the traditional LIBs [3,4].

Despite these advantages, a number of challenges hinder the successful realization of Li/S batteries. Firstly, the electrically insulated sulfur and the deposition of final discharge product Li_2_S result in inefficient cathode material utilization and poor rate capability. Secondly, the high solubility and the shuttle effect of polysulfide intermediates lead to an irreversible capacity loss during charge/discharge cycling. Thirdly, the volumetric changes of the sulfur cathode during the charge/discharge process disrupt the integrity of composite electrode and result in loss of electrical contact [5]. These issues limit the practical utilization of sulfur as an alternative cathode material. Particularly, the volumetric changes, associated with sulfur, cause the shuttle effect and lead to the poor cyclic performance of Li/S batteries [6,7,8].

In order to alleviate the aforementioned issues, various strategies have been proposed. The physical or chemical encapsulation of sulfur into a porous and conductive material is a widely employed approach to circumvent the volumetric changes and enhance the electrical conductivity of composite electrode [9]. Graphene is considered as an ideal sulfur host due to its chemical stability, large surface area, superior electrical conductivity and desirable mechanical properties [10]. Various research groups have shown promising results of sulfur/graphene composite cathodes for lithium/sulfur, however, the cyclic stability is not up to the mark yet. The main problem with graphene/sulfur composites lies with the weak interactions related to physical absorption. Hence, hybrid host structures, such as carbon/polymer composites [11] and carbon/metal oxide composites [12], are designed to enhance the physical interactions between sulfur and host material.

Recently, conducting polymers, such as polypyrrole (PPy), polyacrylonitrile and polythiophene, have garnered significant research attention due to their excellent electrochemical stability and ability to form favorable composite morphologies [13,14,15]. Moreover, owing to the simple synthesis process and the high absorption ability, the PPy is considered as a promising matrix to host sulfur for cathode applications [16].

Herein, we have successfully developed a facile and efficient template-assisted route to synthesize a novel three-dimensional (3D) hierarchical sulfur/polypyrrole/porous graphene (S/PPy/PG) composite, which deliver the excellent electrochemical performances as cathode for Li/S batteries. The study provides a mechanistic insight and opens up avenues for further research in Li/S batteries.

## 2. Materials and Methods

### 2.1. Material Synthesis

**Figure 1:**
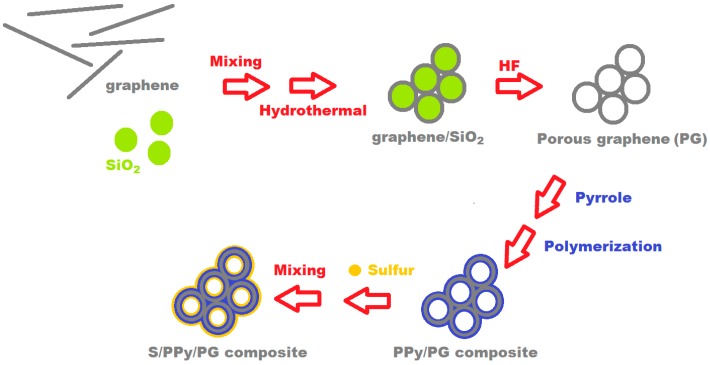
Schematic illustration of S/PPy/PG composite fabrication process.

Graphene was obtained by the modified Hummers method [17]. Porous graphene (PG) was synthesized via a simple hydrothermal method and template etching process, which used commercial silica particles (diameter of about 300 nm) as a hard template. To fabricate PG, 0.5 g of silica particles was immersed in 2.0 g of a homogeneous ethanol solution containing 0.5 g of resol and 0.5 g of triblock copolymer EO_20_PO_70_EO_20_ (P123) for 1 h. The impregnated composites were removed from the solution and kept at room temperature for 6 h, followed by heating at 100 °C for 24 h. The samples were then calcined in N_2_ at 350 °C for 2 h at a heating rate of 1 °C min^−1^ to remove the soft template, and heated further to 900 °C at a rate of 5 °C min^−1^, which was followed by a 2 h soaking for further carbonation. Finally, PG was obtained after the silica particles were removed by washing with HF solution (5 wt. %, 24 h). With FeCl_3_ as an oxidant, the polypyrrole was prepared by using the chemical oxidation method. The method by in-situ polymerization of pyrrole in the presence of porous graphene can be used to prepare the polypyrrole-coated porous graphene (PPy/PG) composite [18]. Figure 1 illustrates the synthesis processs of S/PPy/PG composite schematically.

In a typical reaction, 0.1 g PG was added into a 45 mL mixture with methanol and acetonitrile (1:1 vol) for sonicating for 3 h. After adding 0.3 g pyrrole, the mixture was stirred for 1 h. Subsequently, we have added 20 mL of 0.5 mol L^−1^ FeCl_3_ aqueous solution and the solution sonicated for 3 h. The PPy/PG was obtained by centrifuging at 3000 rpm for 0.5 h. The product was cleaned with deionized water (DI) water and methanol, followed by vacuum drying at 80 °C for 24 h.

To obtain S/PPy/PG composites, 0.3 g PPy/PG was added into 6 g nano-sulfur aqueous suspension (US Nanomaterials, 10 wt. %) in agate milling bowl (50 mL) and mixed well using ball-milling at 800 rpm for 5 h. The ball-milling was performed in a planetary ball mill (QM-3SP04, Nanjing, China) under ambient conditions. Then, the ball-milled suspension was dried at 75 °C for 24 h to remove the residual solvent. Finally, the obtained mixture was heat-treated at 155 °C for 2 h under Ar flow to sulfur be loaded in the PPy/PG composites to form S/PPy/PG.

### 2.2. Material Characterization

The sample was investigated by using Fourier transform infrared spectroscopy (FTIR, TENSOR 27, Bruker Co., Billerica, MA, USA). The field-emission scanning electron microscopy (FE-SEM, S-4800, Hitachi Ltd., Tokyo, Japan) could be used to characterize surface morphology and chemical composition, equipped with an energy dispersive spectrometer (EDS). The transmission electron microscopy (TEM, JEM-2100F, JEOL, Akishima-shi, Tokyo, Japan) could analysis the high-resolution surface structure. The crystalline phases were measured by X-ray diffraction (XRD) analysis (D8 Focus, Bruker, Karlsruhe, Germany). The X-ray photoelectron spectroscopy (XPS, K-Alpha, Thermo Scientific, Waltham, MA, USA) could analysis surface composition. The thermogravimetric analysis (TGA, SDT Q600, TA Instruments-Waters LLC, New Castle, DE, USA) was carried out to assess the weight percentage of components under N_2_ atmosphere in the range of 25–800 °C.

### 2.3. Electrochemical Characterization

The electrochemical performances of the S/PPy/PG cathodes for Li/S batteries were carried out. The electrodes were obtained by mixing the S/PPy/PG composite (80 wt. %), polyvinylidene fluoride (PVDF) binder (10 wt. %) and Ketjen Black (10 wt. %) via using 1-methyl-2-pyrrolidinone (NMP). The homogeneously slurry was pasted onto the carbon-coated aluminum foil and then dried at 60 °C for 12 h. The sulfur loading of ~2 mg cm^−2^ was achieved. The prepared electrode served as a cathode, the pure lithium metal served as the anode and the microporous polypropylene served as a separator. The galvanostatic charge-discharge testing was carried out to assess the Li-storage capacity and cyclic performance of the S/PPy/PG composite. The charge/discharge curves were recorded between 1.5 V and 3.0 V vs. Li/Li^+^. The cyclic voltammetry (CV) was measured with an electrochemical workstation (VersaSTAT 4, Princeton Applied Research, Oak Ridge, TN, USA) at scan rate of 0.1 mV s^−1^.

## 3. Results and Discussion

**Figure 2:**
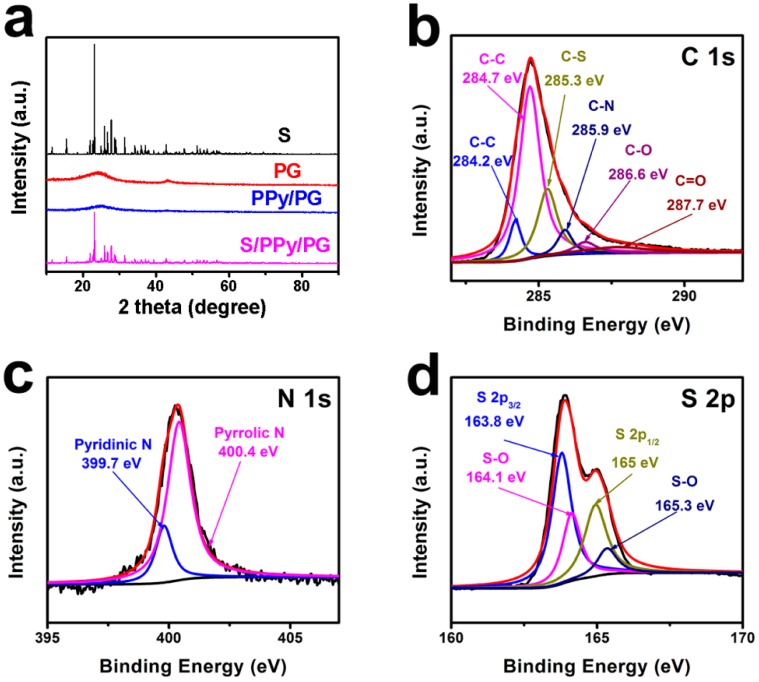
(**a**) XRD patterns of S, PG, PPy/PG and S/PPy/PG composite; high-resolution XPS spectra from core level of (**b**) C 1s, (**c**) N 1s and (**d**) S 2p from S/PPy/PG composite.

Figure 2a delivers the XRD patterns of PG sample has shown typical graphene peaks at 24° and 43°, corresponding to the (002) and (100) planes, respectively [19]. After in-situ polymerization of pyrrole monomer on PG surface and carbonization process, the position of these peaks did not exhibit a significant change, which indicates that the PPy has been uniformly distributed on PG surface. After further sulfur loading, the XRD patterns of S/PPy/PG composite exhibited typical sulfur peaks, indicating that most of the sulfur has been diffused into the cavities of PPy/PG and homogeneously dispersed in fabricated composite [20].

The XPS analysis of S/PPy/PG composites was carried out to confirm the elemental composition and results are shown in Figure 2b–d. In high-resolution C 1s XPS spectra (Figure 2b), the distinct peaks of C–C, C–S, C–N, C–O and C=O are shown. The C–C peak at around 284.2 eV corresponds to the carbon bond in PPy, and the C–C peak located at 284.7 eV confirms the presence of carbon bond in PPy and PG. In addition, the weak intensity ratio of C–O/C–C peaks demonstrates the reduction effect in PG samples [21]. The high-resolution N 1s spectrum (Figure 2c) reveals the pyridinic-N (399.7 eV) and pyrrolic-N (400.4 eV), respectively [22]. In Figure 2d, the peaks at 164.1 and 165.3 eV represent the S–O bonds, indicating a interaction between sulfur and PG in the S/PPy/PG composite. As a result, the nitrogen of PPy, which is coated on the PG surface, and the oxygen-containing functional groups of PG effectively riveted the sulfur onto the surface of S/PPy/PG. Owing to this beneficial phenomenon, the electrochemical performance of S/PPy/PG composite electrodes has been enhanced.

**Figure 3:**
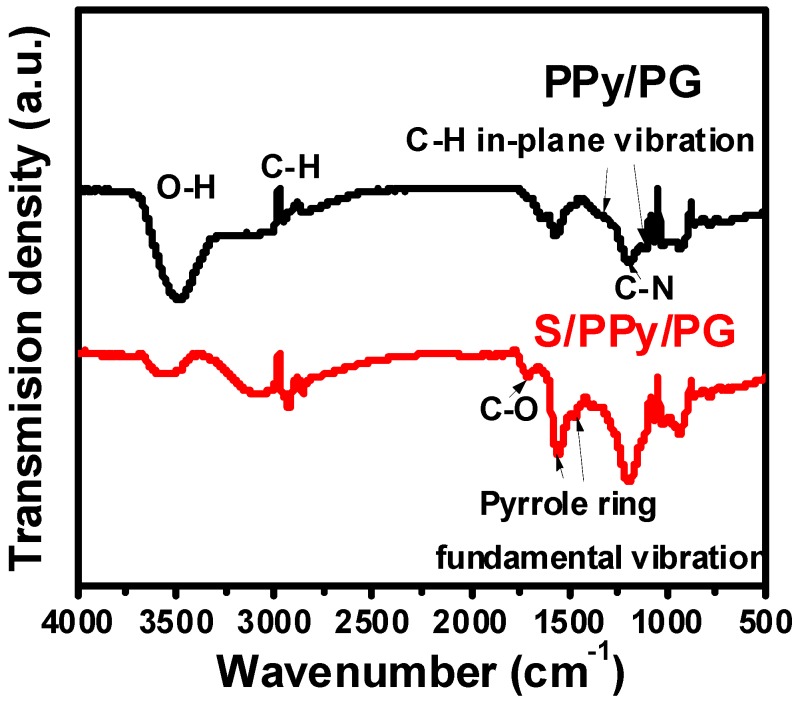
The FTIR spectra of PPy/PG and S/PPy/PG composites.

Figure 3 presents the FTIR spectra of PPy/PG and S/PPy/PG composites. It can be clearly observed that the characteristic bands of the hybrid composites are corresponding to the published literature. The absorption bands, corresponding to O–H stretching at 3500 cm^−1^ and symmetric and asymmetric stretching of C–H at 2920 and 2901 cm^−1^, respectively, confirm the presence of PG in PPy/PG and S/PPy/PG composites. The presence of graphene was observed by C–C skeleton vibration of carbon ring, which has shown a characteristic band at 1562 cm^−1^. The characteristic pyrrole ring vibrations were observed at 1548 cm^−1^ and 1460 cm^−1^. The peaks located at 1092 cm^−1^ and 1308 cm^−1^ correspond to =C–H in-plane vibrations and the peak at 1162 cm^−1^ refers to the characteristic C–N stretching vibrations [23]. In addition, PPy/PG and S/PPy/PG composites have shown that sulfur did not react with PPy and PG during the loading process. The decreased bands intensity in S/PPy/PG composite can be ascribed to the decrease in PPy content. These results confirm that the PPy/PG hybrid architectures have been successfully prepared via in-situ chemical polymerization.

**Figure 4:**
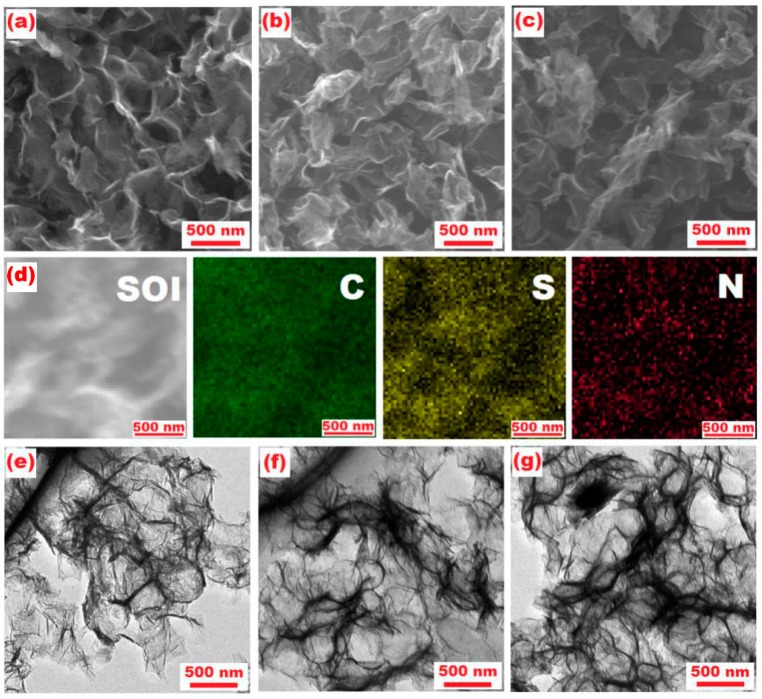
SEM images of (**a**) PG, (**b**) PPy/PG, and (**c**) S/PPy/PG; SEM image of (**d**) S/PPy/PG and corresponding elemental mapping of carbon (C), sulfur (S) and nitrogen (N); TEM images of (**e**) PG, (**f**) PPy/PG and (**g**) S/PPy/PG composite.

As shown in Figure 4a–c, PG exhibits a 3D hierarchical porous structure, which processes a pore diameter of about 300 nm. The adsorption of pyrrole monomers on PG surface resulted from π-π interaction, hydrogen bonding and Van der Waals interactions, which served as an anchor for further polymerization. After sulfur loading, the morphology of PPy/PG did not show a significant change, indicating that a large amount of sulfur has been loaded between the pores of the PPy/PG and a smaller amount has been attached to the surface. The elemental mapping (Figure 4d) confirms the highly uniform dispersion of sulfur, PG and PPy in the S/PPy/PG composites. As shown in the TEM images (Figure 4e,f), the structure and morphology of PPy/PG composites remained same, which indicates that PPy has been formed and fixed on PG surface after polymerization. As sulfur is heavier than graphene and PPy, the darker region in TEM image can be ascribed to sulfur, as shown in Figure 4g. Due to the unique hybrid structure of a highly porous composite, which can provide a large interfacial area between electrode and electrolyte to buffer volumetric changes, the Li/S batteries with S/PPy/PG composite cathodes have exhibited a remarkable rate capacity and excellent cycling stability.

**Figure 5:**
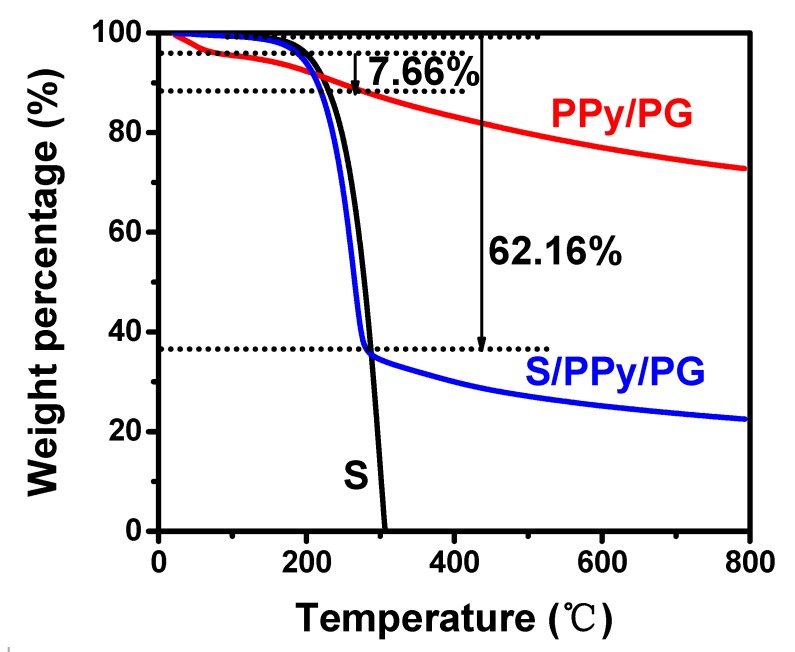
TGA curves of sulfur, PPy/PG and S/PPy/PG composite.

The content of sulfur in the S/PPy/PG composite was estimated by TGA in Figure 5, which had a rapid weight loss during 25–300 °C with a remaining mass of 37.84%, which can be attributed to the loss of S and thermal decomposition of PPy. The weight loss of PPy was 7.66% in the range of 25–300 °C. The sulfur content of S/PPy/PG composite (X) was calculated from the given formula:

62.16% = X% + (100% − X%) × 7.66%

The sulfur loading was found to be 59%.

**Figure 6:**
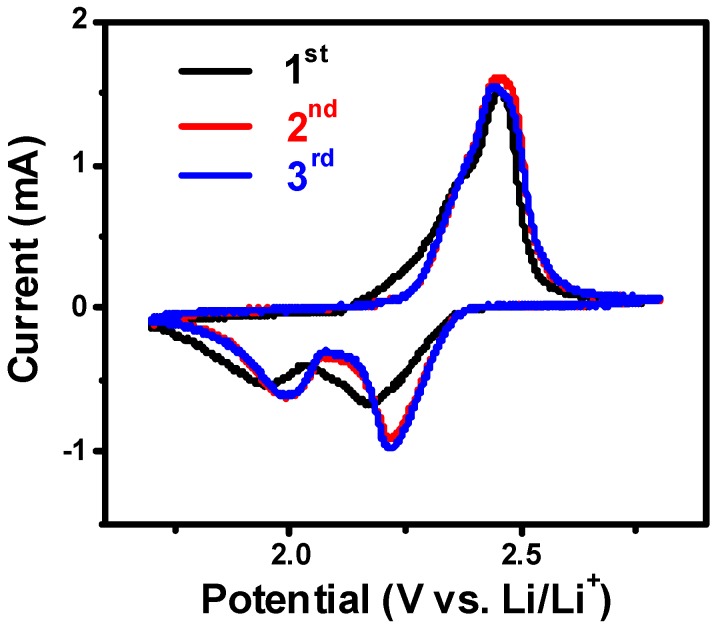
Initial three CV curves of S/PPy/PG composite electrode at a scan rate of 0.1 mV s^−1^.

The initial three CV curves for the S/PPy/PG electrode are shown in Figure 6. During the cathodic sweep, two typical peaks are located at 2.25 and 2.0 V, corresponding to the transformation of sulfur to polysulfides (Li_2_S_n_, 4 ≤ n ≤ 8) and finally to Li_2_S_2_ and Li_2_S, respectively [24]. In the anodic scan, one major peak was observed at about 2.5 V, revealing the oxidation of sulfides to sulfur [25]. We have not observed any peak shift or the emergence of new peaks during further cycling, suggesting excellent electrochemical stability due to the hybrid porous structure of electrode [26].

**Figure 7:**
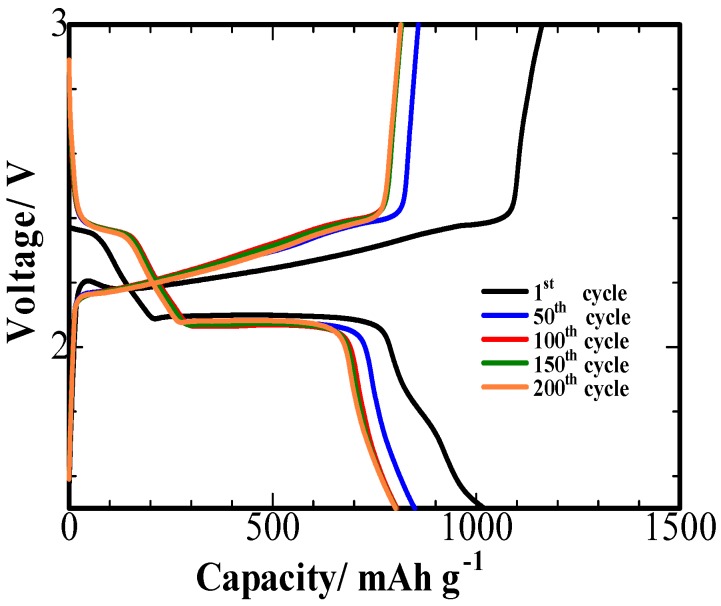
Charge-discharge profiles of S/PPy/PG composite electrode for the 1st, 50th, 100th, 150th and 200th cycles at 0.1 C.

Figure 7 shows the galvanostatic charge-discharge curves of the S/PPy/PG composites at 0.1 C. Two reduction plateaus, consistent with CV curves, were observed in the discharge process. The voltage plateau at 2.3 and 2.0 V are corresponded to the formation of soluble polysulfides and reduction of soluble polysulfides to Li_2_S_2_ or Li_2_S, respectively [27]. In addition, the second flat plateau suggests a uniform formation of Li_2_S [28]. In the 50th, 100th, 150th and 200th cycles, the discharge process exhibits the similar voltage plateaus, which indicates the excellent stability of the fabricated composites.

**Figure 8:**
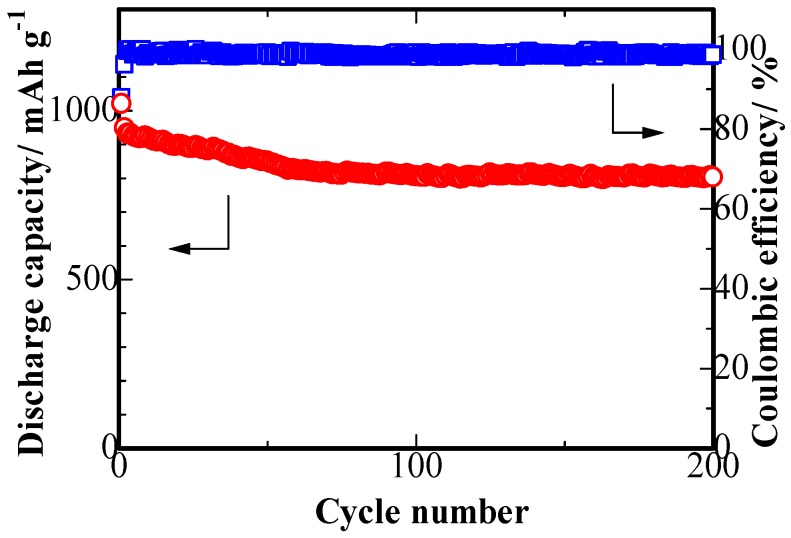
The cyclic performance of S/PPy/PG composite electrode at 0.1 C.

Furthermore, the S/PPy/PG electrodes have demonstrated excellent cycling stability at 0.1 C, and rate performance. Figure 8 presents the cyclic stability of S/PPy/PG electrodes at 0.1 C. The initial specific discharge capacity of 1020 mAh g^−1^ and the capacity retention of 78.6% after 200 cycles are obtained for the S/PPy/PG composite, which corresponds to a low fade rate of 0.1% per cycle. In addition, the coulombic efficiency of S/PPy/PG electrode remained ~100% during charge/discharge cycling, which indicates that the hybrid core-shell and 3D hierarchical porous structure of S/PPy/PG suppresses the shuttle effect.

**Figure 9:**
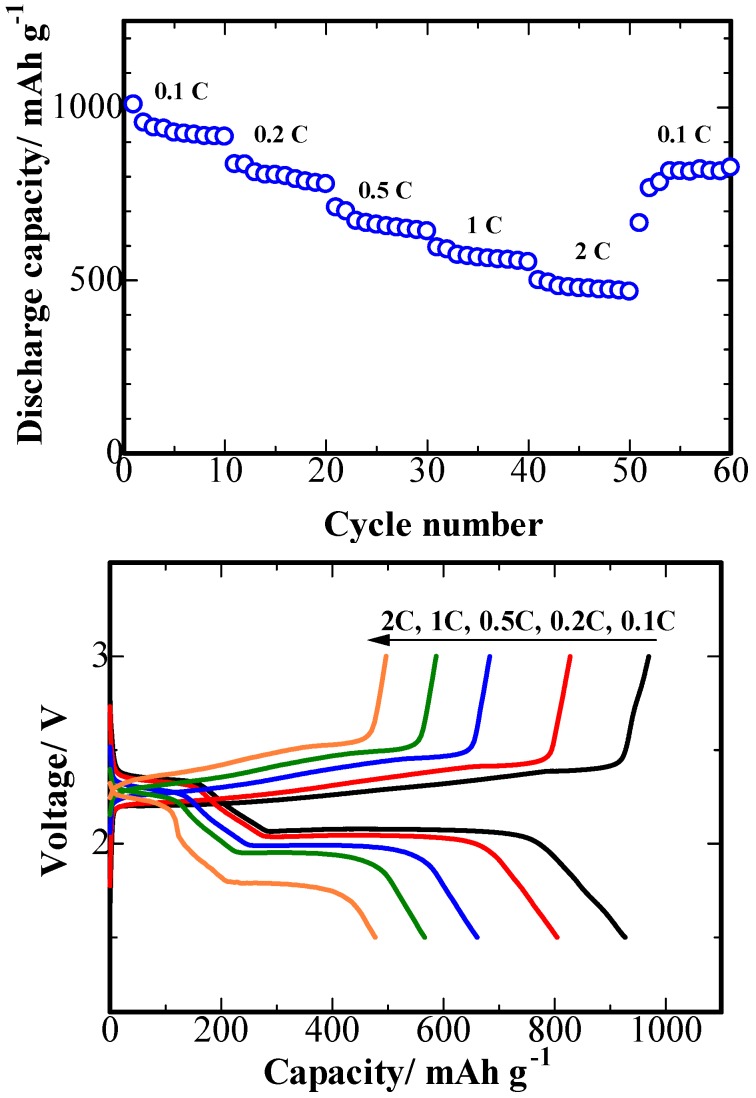
The rate performance of the S/PPy/PG composite electrode, measured by carrying out charge/discharge testing at various current rates.

Figure 9 shows the first ten charge/discharge cycles were carried out at 0.1 C, followed by a stepwise increase towards the higher current densities. The S/PPy/PG composite electrode delivered the specific capacity of 927, 805, 661, 566 and 477 mAh g^−1^ at 0.1, 0.2, 0.5, 1 and 2 C, respectively. Once the current density was returned to the lower value of 0.1 C, the discharge capacity of 816 mAh g^−1^ is recovered, presenting the excellent rate performance. Owing to the hierarchical mesoporous and macroporous structure and enhanced conductivity of S/PPy/PG cathode, the higher capacity, excellent cyclic performance and superior rate capability were realized.

## 4. Conclusions

In summary, we have successfully obtained sulfur impregnated polypyrrole-coated porous graphene (S/PPy/PG) composite, with a 3D hierarchical porous structure, which has demonstrated superior cycling stability and rate performance. The PG sample was synthesized by using template-assisted hydrothermal process and in-situ chemical polymerization of pyrrole monomer was carried out, which resulted in PPy-deposited PG surfaces. Furthermore, the S/PPy/PG composite cathode has shown an excellent cyclic stability, corresponding to a reversible capacity of 802 mAh g^−1^ after 200 cycles. The study provides a novel route to synthesize 3D hierarchical porous network structure and opens up avenues for further research in the area of next-generation Li/S batteries.
